# Safety and efficacy of direct oral anticoagulants in comparison with warfarin across different BMI ranges: A systematic review and meta-analysis

**DOI:** 10.1016/j.amsu.2022.103610

**Published:** 2022-04-14

**Authors:** Talal Almas, Faeez Muhammad, Laiba Siddiqui, Batool Shafi, Rabbia Gul, Rafiya Altaf, Zaeem Abbasi, Ghulam Mustafa, Arham Iqbal, Amatul Rehman Durdana, Maham Dilawar, Adeena Musheer, Kaneez Fatima

**Affiliations:** a Department of Medicine, RCSI University of Medicine and Health Sciences, Dublin, Ireland; b Department of Medicine, Dow University of Health Sciences, Karachi, Pakistan

**Keywords:** DOAC (direct oral anticoagulants), Warfarin, BMI (Body mass index), Safety, Efficacy

## Abstract

**Background:**

Many publications have compared various outcomes defining safety and efficacy of DOACs across different BMI ranges. Our meta-analysis compares warfarin and DOACs for its treatment effects over different BMI ranges.

**Methods:**

A systematic search was conducted from inception to May 2021 on PubMed, Scopus and Embase databases. The data was extracted and pooled using a random effects model. Our study consisted of patients being treated for VTE and AF, across different BMI categories. For the comparison of DOAC, risk ratios (RR) with 95% confidence intervals (CIs) were used, whilst for the second comparison between warfarin and DOACs odds ratios (OR) were used.

**Results:**

In our first comparison, 12 studies (*n* = 254,908 patients) were included. For our second comparison, six studies (*n* = 109,609 patients) were included. Major bleeding events in the underweight group were higher than normal weight [RR: 1.89 (1.10, 3.23); *P* = 0.02; *I*^*2*^ = 0%]. Overweight patients were related with reduced rates of VTE than in patients with normal BMI [RR: 0.86 (0.76, 0.97); *P* = 0.02; *I*^*2*^ = 0%]. In comparison with patients receiving warfarin, DOACs had significantly reduced risk of major bleeding in normal weight, overweight and obese [OR: 0.64 (0.49, 0.83); *P* = 0.0007 *I*^*2*^ = 90%].

**Conclusion:**

The risk of VTE reduces with an increasing BMI, hence there could be a possible obesity paradox in patients with anticoagulation therapy. In comparison to warfarin, DOACs proved to be the safer option by having a reduced risk of bleeding across all BMI categories.

## Highlights

• Warfarin therapy and its effectiveness for prevention of thromboembolism.
• Effect of warfarin and DOACs over different BMI ranges.
• Safety and efficacy of DOACs across different BMI ranges.
• Lower risk of bleeding in normal weight, overweight, and obese patients with DOACs.
• Warfarin and DOACs across different BMI categories in VTE and AF patients.

## 1 Introduction

Venous thromboembolism (VTE) and atrial fibrillation (AF) have a prevalence of approximately 10 million cases annually and a reported 59.7 million cases in 2019 alone, respectively [1,2]. Warfarin therapy has proven its effectiveness in previous years in the prevention of such thromboembolic events [3]. In recent years, the emergence of direct oral anticoagulants (DOACs) has not only shown further efficacy but has overcome several limitations associated with warfarin use [4]. DOAC's offer a further elaborate form of therapy due to their rapid onset of action that does not require bridging with parenteral anticoagulants, along with any dietary restrictions nor continual international normalized ratio (INR) laboratory monitoring [5]. Further advantages of DOAC's include the provision of a large therapeutic window with low drug-drug interactions, predictable pharmacodynamics and the ease of switching patients from low molecular weight heparins (LMWH) and warfarin therapy onto DOAC drug regimens [6].

Despite the wide usage and advantages of DOAC's, there is inadequate evidence in regard to their effect across patients of varying body mass index (BMI) categories. Obesity is associated with a 6.2-fold increased risk for VTE, and is a risk factor for developing AF [7,8]. Thus many of the VTE and AF patients account for obese patients, in addition to patients with a normal BMI. Therefore, because lack of corroboration still remains in the comparison between DOAC's and warfarin therapy for obese patients, further investigation is required. The “obesity paradox” has been demonstrated in several studies in which DOAC's had a greater efficacy in individuals with a higher BMI in comparison to those with a lower BMI [9]. However further analysis on patients of all categories of BMI on different DOACs (apixaban, rivaroxaban, dabigatran and ximelagatran) requires in-depth investigation. Thus, this systematic review and meta-analysis aims to establish better treatment options specific to BMI categories by exploring two comparisons (I) the safety and efficacy of DOAC's in patients with AF or VTE with different BMI categories and (II) the safety and efficacy of DOAC and warfarin in patients with AF or VTE across different BMI categories.

## 2 Methods

This systematic review and meta-analysis was conducted in accordance with PRISMA guidelines [10]. The methods of this study adequately adhere to the AMSTAR checklist [11]. An institutional review board (IRB) approval was not required for this study as the data used is publicly available.

### 2.1 Search strategy

In order to retrieve all relevant articles, a literature review was conducted from inception to May 2021 on PubMed and Scopus using two formulated search strings. The two different search strings were constructed based on the two different criteria of this study, using key terms: atrial fibrillation, venous thromboembolism, deep-vein thrombosis, pulmonary embolism, DOAC, VKA, warfarin, rivaroxaban, apixaban, edoxaban and dabigatran arranged with various configurations. The first search string covered the comparison for the safety and efficacy outcomes of different DOAC's across different BMI categories, in VTE or AF patients. The second search string covered the comparison for the safety and efficacy outcomes of DOACs and warfarin across different BMI categories, in VTE or AF patients. All articles were then transferred to EndNote X7 for the removal of duplicate studies. Two reviewers (FM and LS) screened remaining articles on the basis of title and abstract before conducting a full text screening.

### 2.2 Study selection

Studies were included if the given criteria was fulfilled: (1) observational cohorts (retrospective or prospective) or RCTs; (2) patients with VTE and/or AF across different BMI categories (underweight (<18.5 kg/m^2^), normal weight (18.5–24.9 kg/m^2^), overweight (25.0–29.9 kg/m^2^), obese class I (30.0–34.9 kg/m^2^), obese class II (35.0–39.9 kg/m^2^) and obese class III (≥40 kg/m^2^)), who were ≥18 years old and were treated with one or more specific DOAC mentioned and/or warfarin; (3) demonstrated safety (bleeding) and/or efficacy (stroke/VTE recurrence) outcomes after use of DOAC. Studies were excluded if the given was present: (1) patients with other underlying disorders/risk factors (e.g., diabetes, surgery, cancer) (2) patients with other interventions besides the use of DOAC (e.g., cardioversion, heparin, aspirin).

### 2.3 Data extraction

Members of the review team extracted data based on baseline characteristics and key safety and efficacy outcomes. Outcomes of interest include major bleeding events, and VTE recurrence or stroke. Major bleeding events can be defined as fatal bleeding, bleeding into a critical organ (retroperitoneal, intracranial, intraocular, intraspinal), bleeding requiring surgical revision or angiographic embolization, and bleeding with a fall in hemoglobin of 2 g/dL or documented blood transfusion of 2 or more units according to International Society on Thrombosis and Haemostasis [12]. VTE or stroke was measured based on clinical symptoms.

### 2.4 Statistical analysis

The analysis for the data obtained for both comparisons was done using RevMan 5.4.

Outcomes associated with DOAC therapy across patients with different BMI were pooled using a random effects model. For the dichotomous outcomes comparing each BMI (UW, OW, obese, obese I, obese II-III) with a normal BMI, the risk ratios (RR) and their 95% confidence intervals (CIs) were used. The subgroup analysis compared the different types of DOACs for a specific BMI. Outcomes as a result of DOAC or warfarin therapy in patients with different BMI were also pooled using a random effects model. For the dichotomous outcomes comparing DOAC with warfarin, the odds ratio (OR) and their 95% CIs were used. For both comparisons, to assess heterogeneity, an I2 statistic of >75% reported severe heterogeneity. The subgroup analysis compared the different BMI categories on DOAC or warfarin therapy. A p-value of <0.05 was considered significant. Forest plots visualize the pooled data for both comparisons.

## 3 Results

### 3.1 Literature search and study characteristics

The literature search consisted of two search strings. The first search string, which focused on finding articles for the safety and efficacy of different DOACs across different BMI categories in VTE and AF patients, revealed a total of 2189 studies. Following the exclusion of articles, only 12 met the inclusion criteria. These consisted of 254,908 patients in total who were on one of four DOACs —rivaroxaban, apixaban, dabigatran or ximelagatran. The studies include clinical trials, retrospective, and prospective cohort studies. The second search string focused on related articles for the safety and efficacy of DOACs vs warfarin across different BMI categories in VTE and AF patients and obtained 1561 studies. After the exclusion of several articles, six remained. They contained a total of 109,609 patients undergoing DOAC or warfarin therapy. Three of these articles were cohort studies and the remaining three were randomized clinical trials. Characteristics of each study, relevant to the meta-analysis are described in Table 3 and 4 An overview of the literature search is available in the PRISMA flow charts 1 and 2.

### 3.2 Results of meta-analysis

The results of the meta-analysis of the data extracted from the relevant studies are presented in detailed forest plots in Fig. 1, Fig. 2, Fig. 3, Fig. 4, Fig. 5, Fig. 6, Fig. 7, Fig. 8, Fig. 9, Fig. 10, Fig. 11, Fig. 12.

**Fig. 1:**
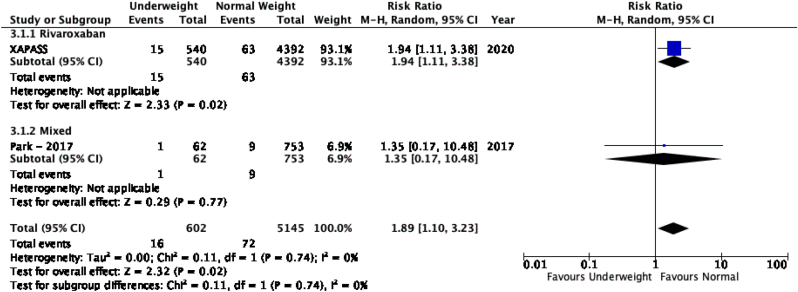
Major bleeding events in underweight patients on different DOACs.

**Fig. 2:**
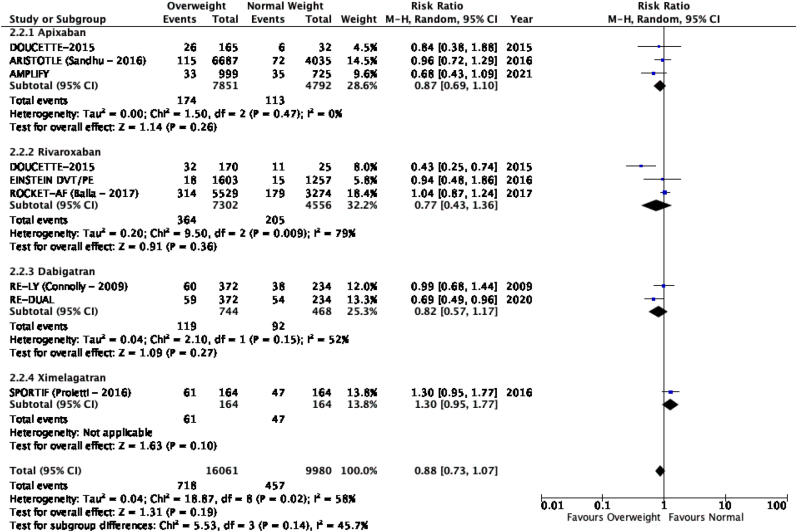
Major bleeding events in overweight patients on different DOACs.

**Fig. 3:**
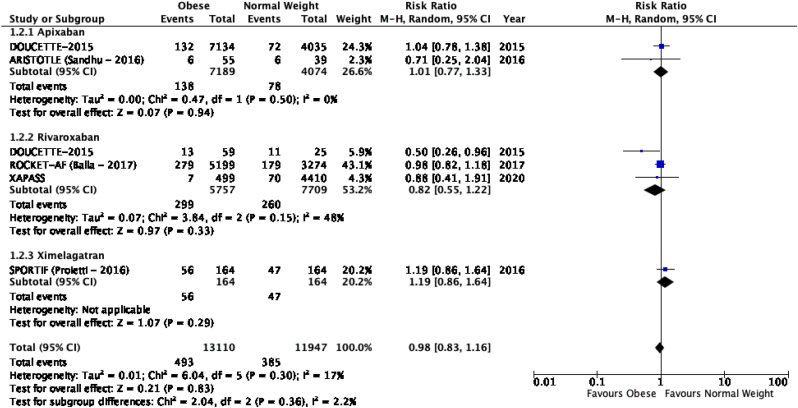
Major bleeding events in obese patients on different DOACs.

**Fig. 4:**
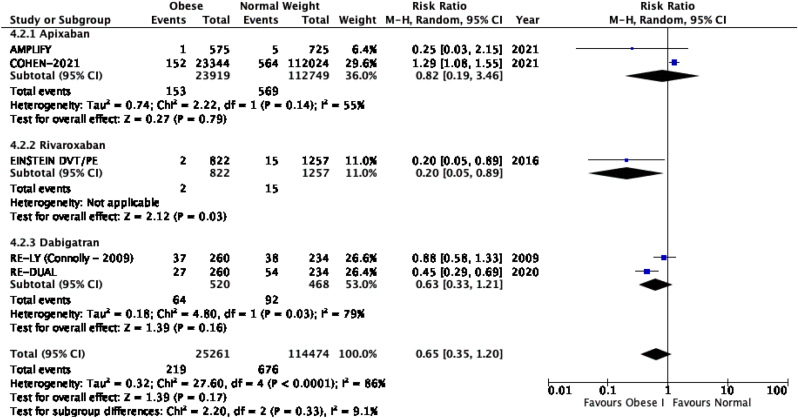
Major bleeding events in obese class I patients on different DOACs.

**Fig. 5:**
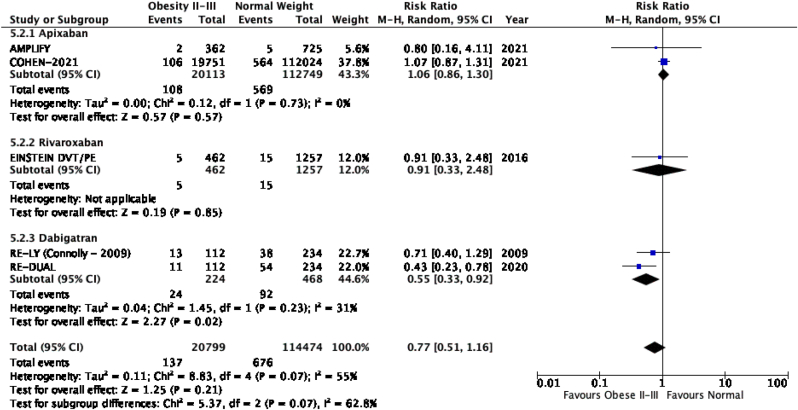
Major bleeding events in obese class II-III patients on different DOACs.

**Fig. 6:**
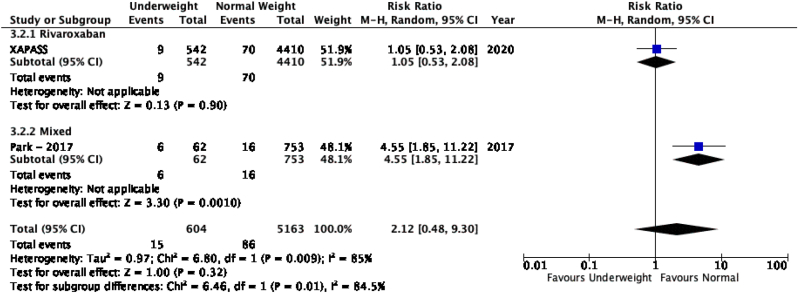
VTE recurrence/stroke in underweight patients on different DOACs.

**Fig. 7:**
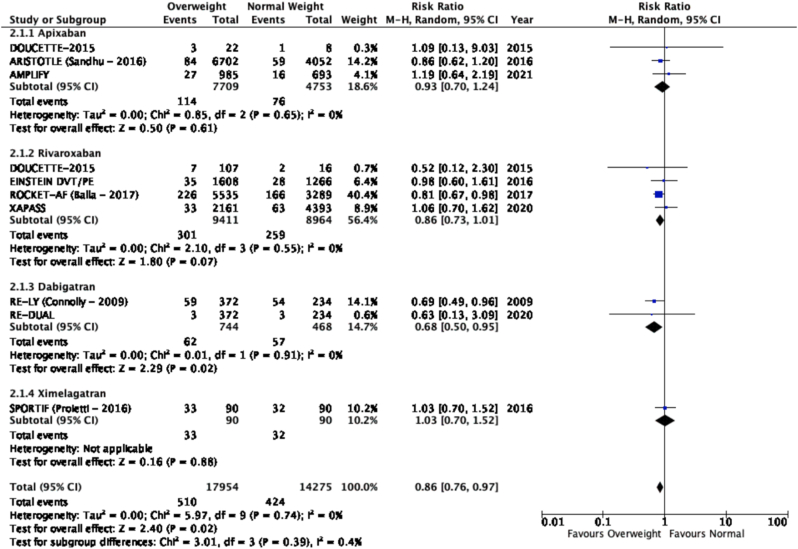
VTE recurrence/stroke in overweight patients on different DOACs.

**Fig. 8:**
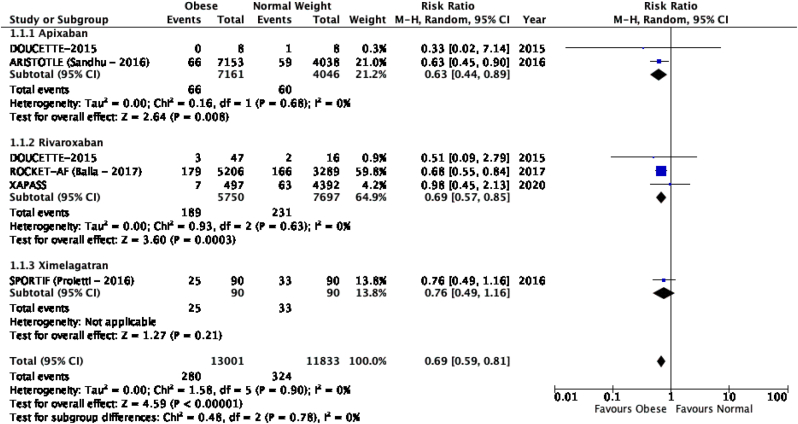
**VTE recurence/stroke in obese patients on different DOAC**s.

**Fig. 9:**
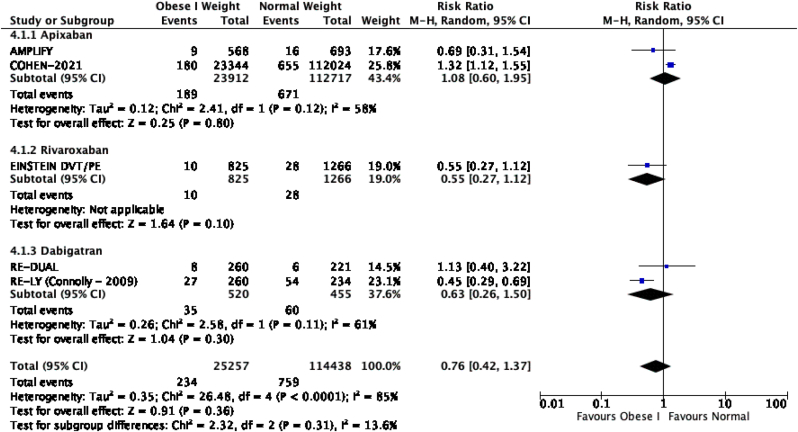
VTE recurrence/stroke in obese class I patients on different DOACs.

**Fig. 10:**
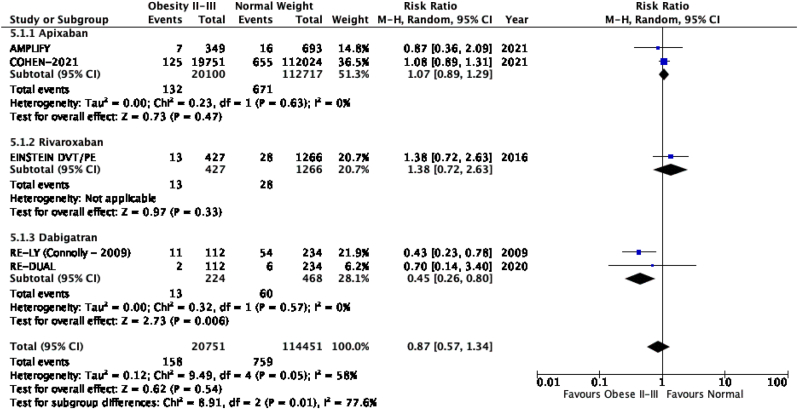
**VTE recurrence/stroke in obese class II-III patients on different DOACs**.

#### 3.2.1 Major bleeding events in patients with different BMIs on DOAC therapy (Fig. 1, Fig. 2, Fig. 3, Fig. 4, Fig. 5)

Major bleeding events in underweight patients were overall significantly higher than in patients with a normal weight [p = 0.02; RR: 1.89 (1.10, 3.23); I^2^ = 0%]. However, events in overweight patients were not significantly different than in patients with a normal weight [p = 0.19; RR: 0.88 (0.73, 1.07); I^2^ = 58%]. Bleeding in obese class I patients was also not significantly different than in patients with a normal BMI [p = 0.83; RR: 0.98 (0.83, 1.16); I^2^ = 17%]. There was no statistical significance in the difference of bleeding events between obese class I patients and patients with a normal weight [p = 0.17; RR: 0.65 (0.35, 1.20); I^2^ = 86%]. Lastly, there was no significant difference in major bleeding events in obese class II-III and normal BMI [p = 0.21; RR: 0.77 (0.51, 1.16); I^2^ = 55%].

#### 3.2.2 VTE recurrence or stroke in patients with different BMIs on DOAC therapy (Fig. 6, Fig. 7, Fig. 8, Fig. 9, Fig. 10)

There was no significant difference for the recurrence of VTE or stroke between underweight and normal weight patients [p = 0.32; RR: 2.12 (0.48, 9.30); I^2^ = 85%], however a subgroup analysis revealed a significantly higher rate of VTE recurrence or stroke in the study with mixed DOAC's (rivaroxaban, apixaban, dabigatran) therapy as compared to the study with only rivaroxaban therapy [p = 0.01; I^2^ = 84.5%]. VTE recurrence in overweight patients was overall significantly lower than in patients with a normal BMI [p = 0.02; RR: 0.86 (0.76, 0.97); I^2^ = 0%]. Obese class I and normal weight patients had no significant difference in recurrence of VTE or stroke [p = 0.35; RR: 0.76 (0.42, 1.37); I^2^ = 85%]. Obese class II-III patients did not have a significant difference in VTE recurrence or stroke when compared to normal weight patients [p = 0.54; RR: 0.87 (0.57, 1.34); I^2^ = 58%], subgroup analysis reported a significant difference in events of VTE recurrence or stroke between studies that used apixaban, rivaroxaban and dabigatran [p = 0.01; I^2^ = 77%].

#### 3.2.3 Major bleeding events with DOAC or warfarin therapy across BMI categories (Fig. 11)

Major bleeding events reported to be significantly higher in patients on warfarin therapy as opposed to DOAC therapy across normal weight, overweight and obese [p = 0.0007; OR: 0.64 (0.49, 0.83); I^2^ = 90%].

**Fig. 11:**
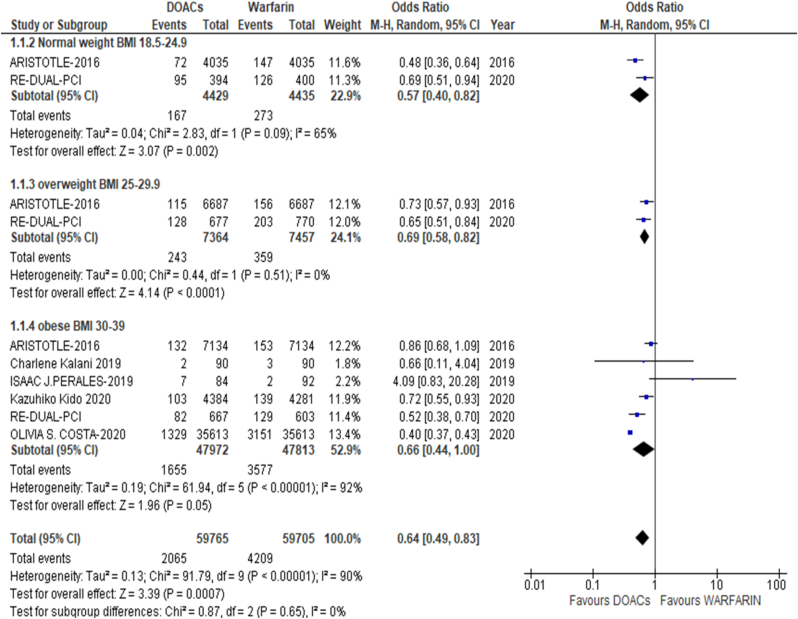
Bleeding in DOAC or warfarin therapy.

#### 3.2.4 VTE recurrence or stroke with DOAC or warfarin therapy across BMI categories (Fig. 12)

There was no significant difference in the recurrence of VTE or stroke in patients using DOAC and warfarin therapy across normal weight, overweight and obese patients [p = 0.96; OR: 1.01 (0.77, 1.31); I^2^ = 90%].

**Fig. 12:**
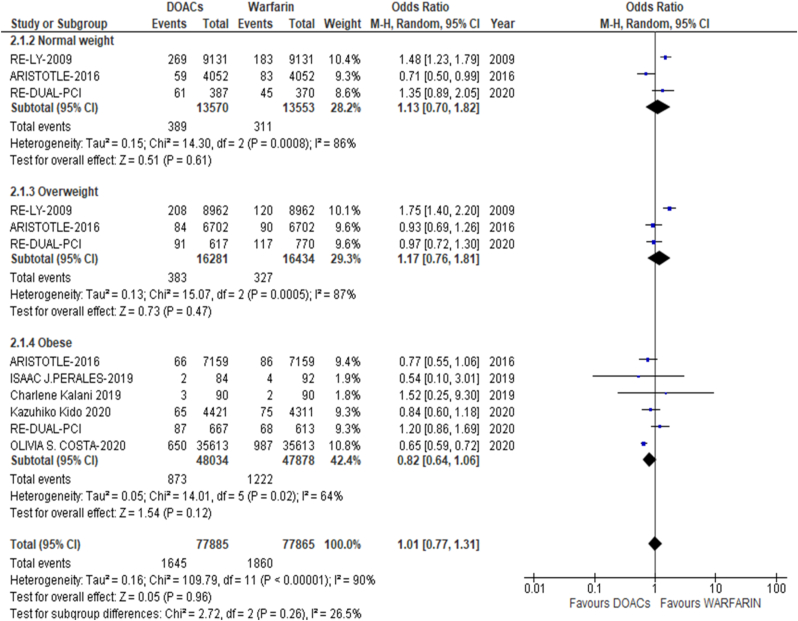
VTE recurrence or stroke in DOAC or warfarin therapy.

## 4 Discussion

The primary findings of this meta-analysis, derived from the plots of major bleeding events and VTE recurrence/stroke across BMI classes, include an increased risk of VTE and bleeding events associated with underweight patients in contrast to normal weight, overweight and obese patients. In the plots comparing DOACs to warfarin, analysis of the safety outcomes signifies an overall reduced risk of bleeding in oral anticoagulants, especially in normal weight patients. In general, an obesity paradox can be interpreted as our results indicate that the efficacy commensurates with the increasing BMI.

Our conclusive findings insinuate that the underweight BMI is more susceptible to high risks of VTE and bleeding when using anticoagulants, which remains consistent with prior meta-analyses [13]. Although more research needs to be done to confirm the hypothesis that being underweight is an independent risk factor for AF, it could still be interpreted by these plausible mechanisms. Firstly, low body weight is directly associated with poor systemic inflammation and endothelial function, which further gives rise to platelet aggregation and adhesion, contributing to embolism and ultimately, death [14,15]. Consequently, patients show an escalation in angiotensin 2 levels, which may cause fibrosis of the heart valve [16]. Another probable theory suggests that stripping away the favorable repercussions adipose tissue possesses causes adiponectin levels to paradoxically rise in lean humans causing the development of AF [17]. An apparent observation in underweight patients is malnourishment which could potentially cause illnesses due to a nutrient and vitamin deficiency [18]. Low body weight patients also show profound effects on heartbeat irregularities leading to significantly greater risk of coronary heart disease, which increases the possibility of AF [19]. Even though we have substantial evidence to authenticate our hypothesis, the studies involved present with questionable reliability. For example, the XAPASS study may have pre-existing bias towards the underweight group developing AF owing to the fact that the patients were older and had lower creatinine clearance levels compared to the other BMI groups. Another contributory cause may be the previously presented medical history of stroke; all these factors contribute to a much higher risk of thromboembolism in AF patients [20]. Drug metabolism also differs from patient to patient due to varying BMIs. The reduced renal and hepatic clearance in UW patients could be a possible explanation for the increased bleeding as they result in a longer half-life of the drug producing a more dangerous impact [21,22]. Due to all of these adverse effects, a rational option would be to decrease dosages of DOACs in UW patients, however reduced doses of medication have shown to have an ineffective impact on the risks of bleeding in multivariate analysis [23].

Our investigation revealed that the associated risk of VTE recurrence/stroke was lower in overweight and obese patients on anticoagulation therapy compared to normal weight. The studies used to derive this data are divided into subgroups by specifically naming the DOACs used; dabigatran shows the most significant advocation of reduced VTE in overweight patients compared to normal weight, whilst rivaroxaban takes the lead in reducing occurrence of VTE in obese patients. Even though a staggering significance is implied for the risks of developing VTE, the small number of studies bring up arguable validity. Moreover, our initial hypothesis of reduced risk of VTE in obese patients is weakened once the insignificant effects of BMI groups on bleeding and risks of adverse effects (incidence of VTE and bleeding) in the divided obese classes (obese classes I, II and III) are taken into consideration. Overall, an anomaly may be observed in obese patients.

The obesity paradox is a commonly seen trend in individual studies with many possible clarifications stating the underlying causes for this contradiction. Firstly, obese patients tend to present with a higher number of comorbidities which increases their requirement for intervention and drug therapies. The medications for these pre-existing diseases could induce a beneficial effect on the cardiovascular system which would decrease the adverse effects triggered by the anti-coagulants [24,25]. Secondly, obesity tends to be correlated with a better metabolic reserve which leads to an increased endurance against the increased metabolic stress that accompanies diseases [26]. Moreover, the fore-mentioned critically high levels of renin-angiotensin II system in underweight patients are reversed in obese patients, hence contributing to safer outcomes [27]. Another defense mechanism for AF patients may be the decreased levels of natriuretic peptide in obese patients which are common promoters of stroke and mortality [28]. Coupled with this mechanism, is the increased production of tumor necrosis factor-α receptors in adipose tissue, which could aid in diffusion of inflammation and arrhythmogenic substrates [29]. Finally, heightened levels of lipoproteins generated by adipose tissue may tie up with the circulating lipopolysaccharides produced during inflammation, cleansing and inhibiting them from stimulating a procoagulant state [30]. Other reasons may include the common fault of conferred selection bias or unmeasured potential risk factors.

Several studies have proposed modifiable factors that exacerbate the adverse effects of obesity paradox in AF patients. For instance, a prior study by Wu et al. [31] reflects an evident obesity paradox in elderly compared to younger patients, which signifies a greater shift with increasing age. The effect of age on the paradox for coronary heart diseases, especially AF is still deemed arguable, hence further investigation is required to confirm this phenomenon. In addition, studies have also shown that patients with comparatively good well-maintained cardiorespiratory fitness suggest advantageous prognosis amongst the coronary heart disease patients. The previous findings demonstrate that cardiorespiratory fitness may also mitigate the obesity paradox in AF considering the observations on coronary heart disease and HF [32,33]. Therefore, indicating that high levels of physical activity is directly associated with decreased risk of AF for all BMI classifications.

Our meta-analysis also investigated the outcomes of warfarin and DOACs in relation to different BMI categories, determining DOACs as the superior treatment. We defined a BMI of 18.5–24.9 kg/m^2 as normal weight and showed that DOACs had significantly more beneficial safety outcomes in normal weight AF patients. On the contrary, obese and overweight patients favored neither, DOACs or warfarin, showing no modifications in their bleeding related outcomes. Overall, the total effect of oral anticoagulants across all BMIs resulted in safer outcomes with reduced risk of bleeding; however, they were non-inferior to warfarin in terms of efficacy. More investigation should be done debating whether warfarin or NVKAs are the better choice for high risk VTE patients. According to the linked studies, DOACs remain to be the preferable choice regardless, due to their rapid onset of action, standard dosages without the need of titration, lack of requirement of routine check-ups, and limited interactions with food and associated drugs [[34], [35], [36], [37], [38], [39], [40], [41], [42], [43], [44], [45], [46], [47], [48], [49], [50], [51], [52], [53], [54], [55], [56], [57]].

### 4.1 Limitations

Many limitations were acknowledged in our study, consequently further studies should take into account these restrictions when collecting data. Primarily, the association between BMI and AF and VTE outcomes may have been modified by several factors involving age, sex, exercise, and cardiorespiratory fitness. Secondly, BMI is not a true measurement of body adiposity hence, taking into consideration the obesity paradox in patients with HF and coronary heart disease, the BMI might not have evaluated the body fat and other body compositions accurately. In the plots involving warfarin, comparison to individual DOACs could not be performed due to limited data available which could have further altered the overall results. Additionally, the safety outcome, all-cause mortality was not included in this study; thus, the connection between BMI and death in patients remains dubious. Secondly, substantial heterogeneity occurred amongst the subgroups (Rivaroxaban trial patients had higher average CHA2DS2-VASc score and mechanisms of Dabigatran may differ in contrast to other DOACs) resulting in inconclusive insignificant results. This warrants future trials to specify the data according to the type of data as well, so the safety and efficacy of the individual DOAC's can be evaluated and assessed under variety of clinical settings. The data collected for the meta-analysis did not provide measurements of activated factor X levels, thus it is not evaluated by the study and could possibly affect the outcomes. The study does not evaluate the different dosages given to different groups of BMI. Furthermore, our results are regarded as hypothesis generating as they are based on post hoc analysis such as RCTs and observational studies. Finally, we were unable to assess the effect of preceding comorbidities (cancer or chronic renal disease) or pre-diagnosed medications (cardiac associated or anti diabetic) on AF patients due to the lack of extensive statistics.

## 5 Conclusion

In conclusion, our study demonstrates that a reduced risk of VTE is associated with an increasing BMI. There could be a possible obesity/lean paradox in AF patients with anticoagulation therapy but follow-up studies should ensure validation before prescribing medication. Finally, the safety outcome, across diverse BMI, of DOACs proved to be more favorable than warfarin in normal weight, overweight, and obese patients.

## Ethical approval

N/A.

## Sources of funding

None to declare.

## Author contributions

Talal Almas - Conceptualization and Designing Study.

Faeez Muhammad – Into and Methods (Manuscript writing), Data Collection, Baseline Characteristics, Forest Plots (DOAC vs Warfarin)

Laiba Siddiqui – Intro and Methods (Manuscript Writing), Data Collection.

Batool Shafi – Forest Plots (DOAC for different BMIs), Data Collection.

Rabbia Gul – Manuscript writing (Abstract, Discussion, Limitations, Conclusion), Data Collection.

Rafiya Altaf - Manuscript writing (Abstract, Discussion, Limitations, Conclusion), Data Collection.

Zaeem Abbasi – Forest Plots (DOAC vs Warfarin), Data Collection.

Ghulam Mustafa - Forest Plots (DOAC vs Warfarin), Data Collection.

Arham Iqbal - Forest Plots (DOAC vs Warfarin), Data Collection.

Amatul Rehman Durdana – Manuscript editing, Data Collection.

Maham Dilawar - Forest Plots (DOAC for different BMIs), Data Collection.

Adeena Musheer – Manuscript editing, formatting.

Kaneez Fatima - Conceptualization and Designing Study.

## Registration of research studies

1. Name of the registry:

2. Unique Identifying number or registration ID:

3. Hyperlink to your specific registration (must be publicly accessible and will be checked):

## Guarantor

Batool Shafi - batoolshafi@gmail.com.mailto:batoolshafi@gmail.com

## Consent

N/A.

## Provenance and peer review

Not commissioned, externally peer-reviewed.

## Declaration of competing interest

None to declare.
