# Supplementary material for: Safety and efficacy of direct oral anticoagulants in comparison with warfarin across different BMI ranges: A systematic review and meta-analysis
Source: Ann Med Surg (Lond). 2022 Apr 14;77:103610. doi: 10.1016/j.amsu.2022.103610 (PMC9142411; doi:10.1016/j.amsu.2022.103610)

# SUPPLEMENTARY MATERIAL

# Supplemental Table 1. Search strategy used in each database searched (for DOAC across BMI).

| PubMed(2172 results) | ((("direct"[All Fields] OR "directed"[All Fields] OR "directing"[All Fields] OR "direction"[All Fields] OR "directional"[All Fields] OR "directions"[All Fields] OR "directivities"[All Fields] OR "directivity"[All Fields] OR "directs"[All Fields]) AND ("mouth"[MeSH Terms] OR "mouth"[All Fields] OR "oral"[All Fields]) AND ("anticoagulants"[Pharmacological Action] OR "anticoagulants"[MeSH Terms] OR "anticoagulants"[All Fields] OR "anticoagulant"[All Fields] OR "anticoagulate"[All Fields] OR "anticoagulated"[All Fields] OR "anticoagulating"[All Fields] OR "anticoagulation"[All Fields] OR "anticoagulations"[All Fields] OR "anticoagulative"[All Fields])) OR "DOAC"[All Fields] OR ("dabigatran"[MeSH Terms] OR "dabigatran"[All Fields] OR "dabigatran s"[All Fields]) OR ("dabigatran"[MeSH Terms] OR "dabigatran"[All Fields] OR "pradaxa"[All Fields] OR ("dabigatran"[All Fields] AND "etexilate"[All Fields]) OR "dabigatran etexilate"[All Fields]) OR ("rivaroxaban"[MeSH Terms] OR "rivaroxaban"[All Fields]) OR ("rivaroxaban"[MeSH Terms] OR "rivaroxaban"[All Fields] OR "xarelto"[All Fields]) OR ("apixaban"[Supplementary Concept] OR "apixaban"[All Fields] OR "apixaban s"[All Fields]) OR ("apixaban"[Supplementary Concept] OR "apixaban"[All Fields] OR "eliquis"[All Fields] OR "apixaban s"[All Fields]) OR ("edoxaban"[Supplementary Concept] OR "edoxaban"[All Fields]) OR ("edoxaban"[Supplementary Concept] OR "edoxaban"[All Fields] OR "savaysa"[All Fields]) OR ("betrixaban"[Supplementary Concept] OR "betrixaban"[All Fields]) OR ("betrixaban"[Supplementary Concept] OR "betrixaban"[All Fields] OR "bevyxxa"[All Fields])) AND ("BMI"[All Fields] OR ("body mass index"[MeSH Terms] OR ("body"[All Fields] AND "mass"[All Fields] AND "index"[All Fields]) OR "body mass index"[All Fields]) OR ("body mass index"[MeSH Terms] OR ("body"[All Fields] AND "mass"[All Fields] AND "index"[All Fields]) OR "body mass index"[All Fields] OR ("quetelet"[All Fields] AND "index"[All Fields]) OR "quetelet index"[All Fields]) OR ("weight s"[All Fields] OR "weighted"[All Fields] OR "weighting"[All Fields] OR "weightings"[All Fields] OR "weights and measures"[MeSH Terms] OR ("weights"[All Fields] AND "measures"[All Fields]) OR "weights and measures"[All Fields] OR "weight"[All Fields] OR "body weight"[MeSH Terms] OR ("body"[All Fields] AND "weight"[All Fields]) OR "body weight"[All Fields] OR "weights"[All Fields])) |
| --- | --- |
| Scopus(304 results) | **(direct oral anticoagulant OR DOAC OR dabigatran OR Pradaxa OR rivaroxaban OR Xarelto OR apixaban OR Eliquis OR edoxaban OR Savaysa OR betrixaban OR Bevyxxa) AND (BMI OR body mass index OR quetelet index OR weight)** |

**Supplemental Table 2. Search strategy used in each database searched (for DOAC vs WARFARIN).**

| PubMed  (1,618 results) | ((("direct"[All Fields] OR "directed"[All Fields] OR "directing"[All Fields] OR "direction"[All Fields] OR "directional"[All Fields] OR "directions"[All Fields] OR "directivities"[All Fields] OR "directivity"[All Fields] OR "directs"[All Fields]) AND ("mouth"[MeSH Terms] OR "mouth"[All Fields] OR "oral"[All Fields]) AND ("anticoagulants"[Pharmacological Action] OR "anticoagulants"[MeSH Terms] OR "anticoagulants"[All Fields] OR "anticoagulant"[All Fields] OR "anticoagulate"[All Fields] OR "anticoagulated"[All Fields] OR "anticoagulating"[All Fields] OR "anticoagulation"[All Fields] OR "anticoagulations"[All Fields] OR "anticoagulative"[All Fields])) OR "DOAC"[All Fields] OR ("New"[All Fields] AND ("mouth"[MeSH Terms] OR "mouth"[All Fields] OR "oral"[All Fields]) AND ("anticoagulants"[Pharmacological Action] OR "anticoagulants"[MeSH Terms] OR "anticoagulants"[All Fields] OR "anticoagulant"[All Fields] OR "anticoagulate"[All Fields] OR "anticoagulated"[All Fields] OR "anticoagulating"[All Fields] OR "anticoagulation"[All Fields] OR "anticoagulations"[All Fields] OR "anticoagulative"[All Fields])) OR ("n 4 oleylcytosine arabinoside"[Supplementary Concept] OR "n 4 oleylcytosine arabinoside"[All Fields] OR "noac"[All Fields]) OR ("dabigatran"[MeSH Terms] OR "dabigatran"[All Fields] OR "dabigatran s"[All Fields]) OR ("dabigatran"[MeSH Terms] OR "dabigatran"[All Fields] OR "pradaxa"[All Fields] OR ("dabigatran"[All Fields] AND "etexilate"[All Fields]) OR "dabigatran etexilate"[All Fields]) OR ("rivaroxaban"[MeSH Terms] OR "rivaroxaban"[All Fields]) OR ("rivaroxaban"[MeSH Terms] OR "rivaroxaban"[All Fields] OR "xarelto"[All Fields]) OR ("apixaban"[Supplementary Concept] OR "apixaban"[All Fields] OR "apixaban s"[All Fields]) OR ("apixaban"[Supplementary Concept] OR "apixaban"[All Fields] OR "eliquis"[All Fields] OR "apixaban s"[All Fields]) OR ("edoxaban"[Supplementary Concept] OR "edoxaban"[All Fields]) OR ("edoxaban"[Supplementary Concept] OR "edoxaban"[All Fields] OR "savaysa"[All Fields]) OR ("betrixaban"[Supplementary Concept] OR "betrixaban"[All Fields]) OR ("betrixaban"[Supplementary Concept] OR "betrixaban"[All Fields] OR "bevyxxa"[All Fields])) AND ("warfarin"[MeSH Terms] OR "warfarin"[All Fields] OR "warfarin s"[All Fields] OR "warfarinization"[All Fields] OR "warfarinized"[All Fields] OR "warfarins"[All Fields] OR ("warfarin"[MeSH Terms] OR "warfarin"[All Fields] OR "coumadin"[All Fields] OR "warfarin s"[All Fields] OR "warfarinization"[All Fields] OR "warfarinized"[All Fields] OR "warfarins"[All Fields]) OR ("jantoven"[All Fields] OR "warfarin"[MeSH Terms] OR "warfarin"[All Fields] OR "warfarin s"[All Fields] OR "warfarinization"[All Fields] OR "warfarinized"[All Fields] OR "warfarins"[All Fields]) OR ("controling"[All Fields] OR "controllability"[All Fields] OR "controllable"[All Fields] OR "controllably"[All Fields] OR "controller"[All Fields] OR "controller s"[All Fields] OR "controllers"[All Fields] OR "controlling"[All Fields] OR "controls"[All Fields] OR "prevention and control"[MeSH Subheading] OR ("prevention"[All Fields] AND "control"[All Fields]) OR "prevention and control"[All Fields] OR "control"[All Fields] OR "control groups"[MeSH Terms] OR ("control"[All Fields] AND "groups"[All Fields]) OR "control groups"[All Fields])) AND ("BMI"[All Fields] OR ("body mass index"[MeSH Terms] OR ("body"[All Fields] AND "mass"[All Fields] AND "index"[All Fields]) OR "body mass index"[All Fields]) OR ("body mass index"[MeSH Terms] OR ("body"[All Fields] AND "mass"[All Fields] AND "index"[All Fields]) OR "body mass index"[All Fields] OR ("quetelet"[All Fields] AND "index"[All Fields]) OR "quetelet index"[All Fields]) OR ("weight s"[All Fields] OR "weighted"[All Fields] OR "weighting"[All Fields] OR "weightings"[All Fields] OR "weights and measures"[MeSH Terms] OR ("weights"[All Fields] AND "measures"[All Fields]) OR "weights and measures"[All Fields] OR "weight"[All Fields] OR "body weight"[MeSH Terms] OR ("body"[All Fields] AND "weight"[All Fields]) OR "body weight"[All Fields] OR "weights"[All Fields])) |
| --- | --- |
| Scopus  (14 results) | (Direct oral anticoagulant OR DOAC OR New Oral Anticoagulants OR NOAC OR dabigatran OR pradaxa OR rivaroxaban OR xarelto OR apixaban OR eliquis OR edoxaban OR savaysa OR betrixaban OR bevyxxa) AND (warfarin OR coumadin OR jantoven OR control) AND (BMI OR body mass index OR quetelet index OR weight) |

**Supplemental Table 3. Baseline characteristics of included patients (for DOAC across BMI).**

| Study | Design | Total Patients | Percentage of males | Mean age (years) | Underlying pathology | Anticoagulants | BMI < 18.5  (underweight) | BMI 18.5-24.9  (Normal weight) | BMI 25.0-29.9  (Overweight) | BMI ≥ 30.0  (Obese) | Follow up time |
| --- | --- | --- | --- | --- | --- | --- | --- | --- | --- | --- | --- |
| RE-LY, 2009 | Randomized control trial | 18,113 | 63.6% | 71 | AF | Dabigatran and Warfarin | - | - | 9,131 (<28 BMI) | 8962 (≥28 BMI) | 2.0 years (median) |
| SPORTIF, 2016 | Clinical trial | 3,651 | 69.9% | 72 | NVAF | Ximelgatran, warfarin | - | 874 | 1446 | 1310 | 567 days (median) |
| ARISTOTLE, 2016 | Randomized clinical trial | 17,193 | 67.6% | 69 | AF | Apixaban, warfarin | 194 (excluded from study) | 4,052 | 6702 | 7159 | 1.8 years (median) |
| EINSTEIN DVT/PE, 2016 | Subanalysis | 8,230 | 54.5% | - | VTE (DVT/PE) | Rivaroxaban . enoxaparin, VKA (warfarin or acenocoumarol | - | 2,481 (<25 BMI) | 3,258 | 115 | ≤12 months |
| ROCKET AF, 2017 | Post hoc analysis | 14,030 | 60.7% | - | AF | Rivaroxaban, warfarin | 141 (excluded from study) | 3,289 | 5,535 | 5,206 | 2 years |
| PARK, 2017 | Retrospective cohort | 1,353 | 59.8% | 72.6 ± 8.9 | AF | Dabigatran, rivaroxaban, apixaban | 62 | 753 | 538 | - | 7 months |
| ENGAGE AF, 2019 | Randomized clinical trial | 21,105 | 62.1% | 71 | AF | Warfarin& Edoxaban | 177 | 4491 | 7903 | 8457 | 2.8 Years |
| RE-DUAL, 2020 | Randomized clinical trial | 2,721 | 23.9% | - | NVAF | Dabigatran, warfarin | - | 618 (patients were <25 BMI) | 1113 | 990 | ≥ 6 months |
| XAPASS, 2020 | Prospective cohort | 7618 | 62.5% | 75 | NVAF | Rivaroxaban | 542 | 4410 | 2167 | 499 | 1 year |
| DOUCETTE,2020 | Retrospective Cohort | 398 | 53 % | AF (72 ± 12)  VTE (65 ± 17) | NVAF &  VTE | Rivaroxaban  Apixaban, Dabigatran | 3 | 84 | 116 | 193 | ≤ 2.5 Years |
| COHEN, 2021 | Real world study | 155,119 | 45.2% | 65 | VTE | Warfarin & Apixaban | - | - | 112,024  (Patients were <30 BMI) | 43,095 | 6 months |
| AMPLIFY, 2021 | Post HOC analysis | 5359 | 58.7% | - | VTE | Apixaban, enoxaparin, warfarin |  | 1442 (≤25 BMI) | 2,045 | 1,872 | - |

**Supplemental Table 4. Baseline characteristics of included patients (for DOAC vs WARFARIN).**

| Study | Design | Total patients | Percentage of males | Mean age (years) | Underlying pathology | Anticoagulants | BMI < 18.5  (underweight) | BMI 18.5-24.9  (Normal weight) | BMI 25.0-29.9  (Overweight) | BMI ≥ 30.0  (Obese) | Follow up time |
| --- | --- | --- | --- | --- | --- | --- | --- | --- | --- | --- | --- |
| RE-LY, 2009 | Randomized clinical trial | 18,113 | 63.6% | 71 | AF | Dabigatran and Warfarin | - | - | 9,131 (<28 BMI) | 8962 (≥28 BMI) | 2.0 years (median) |
| ARISTOTLE, 2016 | Randomized clinical trial | 17,193 | 67.6% | 69 | AF | Apixaban, warfarin | 194 (excluded from study) | 4,052 | 6702 | 7159 | 1.8 years (median) |
| charles Kalani, 2019 | Retrospective cohort study | 180 | 58.3% | 62 | AF | Apixaban, rivaroxaban, dabigatran, warfarin | - | - | - | 180 | - |
| isaac J.perales, 2019 | Retrospective cohort study | 176 | 54% | 56 ± 14.5 | AF and VTE | Rivaroxaban, warfarin | - | - | - | 176 | 1 year |
| RE-DUAL, 2020 | Randomized clinical trial | 2,721 | 23.9% | - | NVAF | Dabigatran, warfarin | - | 618 (patients were <25) | 1113 | 990 | ≥ 6 months |
| olivia S Costa 2020 | Cohort analysis | 71,226 | 60.1% | 68 | NVAF | Rivaroxaban, warfarin | - | - | - | 71,262 | 2 ½ years (mean) |

Note:

- Patients were only eligible if their BMI was available.

## **PRISMA FLOW CHART 1 (DOAC across BMI)**


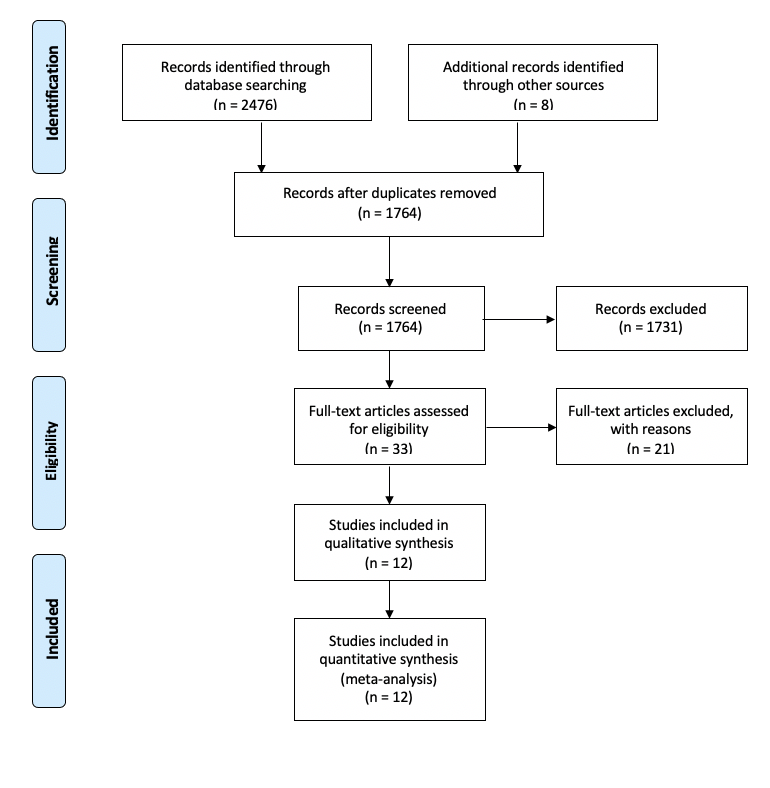


**PRISMA FLOW CHART 2 (DOAC vs WARFARIN)**
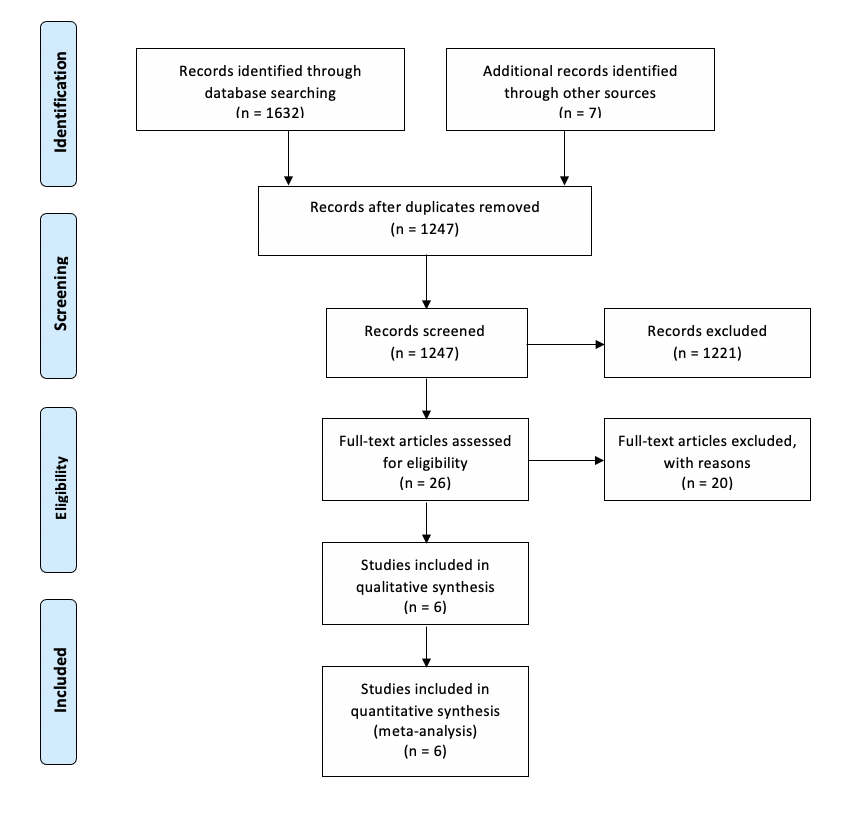

Supplement: Multimedia component 3 [file mmc3.docx]
